# Neorogioltriol and Related Diterpenes from the Red Alga *Laurencia* Inhibit Inflammatory Bowel Disease in Mice by Suppressing M1 and Promoting M2-Like Macrophage Responses

**DOI:** 10.3390/md17020097

**Published:** 2019-02-02

**Authors:** Maria G. Daskalaki, Dimitra Vyrla, Maria Harizani, Christina Doxaki, Aristides G. Eliopoulos, Vassilios Roussis, Efstathia Ioannou, Christos Tsatsanis, Sotirios C. Kampranis

**Affiliations:** 1 Laboratory of Biochemistry, School of Medicine, University of Crete, Heraklion 70013, Greece; m.daskalaki@med.uoc.gr; 2 Laboratory of Clinical Chemistry, School of Medicine, University of Crete, Heraklion 70013, Greece; cdoxaki@med.uoc.gr; 3 Institute of Molecular Biology and Biotechnology, FORTH, Heraklion, Crete 71110, Greece; di_micro@hotmail.com (D.V.); eliopag@med.uoa.gr (A.G.E.); 4 Section of Pharmacognosy and Chemistry of Natural Products, Department of Pharmacy, National and Kapodistrian University of Athens, Panepistimiopolis Zografou, Athens 15771, Greece; mariachariz@pharm.uoa.gr (M.H.); roussis@pharm.uoa.gr (V.R.); 5 Present address: Laboratory of Biology, School of Medicine, National and Kapodistrian University of Athens, Athens 11527, Greece; 6 Present address: Section of Plant Biochemistry, Department of Plant and Environmental Sciences, University of Copenhagen, Thorvaldsensvej 40, 1871 Frederiksberg C, Denmark

**Keywords:** *Laurencia*, halogenated diterpenes, neorogioltriol, cytokine, nitric oxide, TNF-alpha, colitis

## Abstract

Macrophages are central mediators of inflammation, orchestrating the inflammatory response through the production of cytokines and nitric oxide. Macrophages obtain pro-inflammatory (M1) and anti-inflammatory (M2) phenotypes, which can be modulated by soluble factors, including natural products. Despite the crucial protective role of inflammation, chronic or deregulated inflammation can lead to pathological states, such as autoimmune diseases, metabolic disorders, cardiovascular diseases, and cancer. In this case, we studied the anti-inflammatory activity of neorogioltriol (**1**) in depth and identified two structurally related diterpenes, neorogioldiol (**2**), and *O*^11^,15-cyclo-14-bromo-14,15-dihydrorogiol-3,11-diol (**3**), with equally potent activity. We investigated the mechanism of action of metabolites **1**–**3** and found that all three suppressed macrophage activation and promoted an M2-like anti-inflammatory phenotype by inducing expression of Arginase1, MRC1, IRAK-M, the transcription factor C/EBPβ, and the miRNA miR-146a. In addition, they suppressed iNOS induction and nitric oxide production. Importantly, treatment of mice with **2** or **3** suppressed DSS-induced colitis by reducing tissue damage and pro-inflammatory cytokine production. Thus, all these three diterpenes are promising lead molecules for the development of anti-inflammatory agents targeting macrophage polarization mechanisms.

## 1. Introduction

Inflammation is a highly homeostatic defense mechanism triggered by damaged cells, irritants, and pathogens, which result in a cascade of events involving the secretion of inflammatory cytokines and activation and differentiation of immune cells. Transcriptional and post transcriptional regulation of the inflammatory response ensures its coordinated fundamental protective role. However, a deregulated response results in chronic inflammation, which causes pathological states, such as autoimmune diseases, cardiovascular diseases, metabolic disorders, diabetes, and cancer [1].

The magnitude and type of inflammatory response is regulated by cytokines, signaling molecules, and miRNAs [2], which are induced by the inflammatory trigger, but are also affected by external factors, such as natural products possessing immunomodulatory properties. Macrophages play a central role in the initiation and resolution of the inflammation responding to inflammatory signals and triggering T helper cell responses. Macrophages are found in all tissues displaying high functional plasticity in response to the microenvironment and extracellular stimuli. Depending on the environmental stimuli and stage of activation, macrophages obtain different activation phenotypes broadly defined as classically (M1) or alternatively activated (M2), which include an array of M-like phenotypes [3]. Classically activated macrophages (M1) are generated in response to LPS and/or IFN-γ, trigger Th1 mediated responses, and result in the expression of pro-inflammatory cytokines, such as TNFα, IL-6, IL-12, and the production of nitric oxide (NO). Alternatively, activated macrophages (M2) are generated in response to cytokines IL-4, IL-10, immunocomplexes, and glucocorticoids, mediate Th2 responses, and are characterized by the expression of resolution factors, such as IL-10, Arginase 1, MRC1, Ym1, and Fizz1. M2 macrophages play a central role in the healing process and characterize endotoxin tolerance [4].

Previously, we reported the isolation of the brominated diterpene neorogioltriol (**1**) from the organic extract of the red alga *Laurencia glandulifera* and showed that it possesses potent analgesic and anti-inflammatory activities [5]. Subsequently, we showed that **1** reduces NF-κΒ and COX-2 levels in an in vitro model [6]. In view of the increasing need to fight chronic inflammatory diseases and the urgent need for the discovery of new and efficient therapeutic molecules, we aimed to further illuminate the function of neorogioltriol (**1**) in inflammation. In this report, we studied the anti-inflammatory mechanism of **1** using both in vivo and in vitro inflammatory models and identified two additional related metabolites known as neorogioldiol (**2**) and *O*^11^,15-cyclo-14-bromo-14,15-dihydrorogiol-3,11-diol (**3**) with equally potent anti-inflammatory activity [7].

## 2. Results and Discussion

### 2.1. Isolation of Metabolites **1**–**3** and Evaluation of their Anti-Inflammatory Capacity

**Figure 1:**
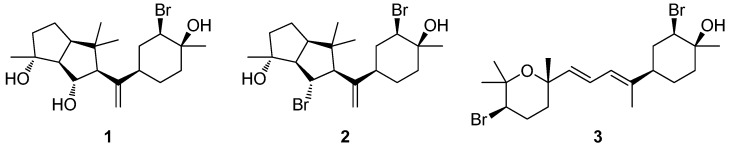
Neorogioltriol (**1**), neorogioldiol (**2**), and *O*^11^,15-cyclo-14-bromo-14,15-dihydrorogiol-3,11-diol (**3**) isolated from *Laurencia* sp. collected from Vatsa bay in Kefalonia island.

Attempts to re-isolate additional amounts of neorogioltriol (**1**) in order to proceed with the elucidation of its mechanism of action proved rather unsuccessful since **1** could not be traced for several seasons in the population of *L. glandulifera* growing in Argostoli bay or in Kefalonia Island, which was initially used as source material for its isolation. Trying to tackle this problem, we extended our search in populations of *Laurencia* spp. collected from nearby locations and, in order to overcome the time-consuming phytochemical analysis, we developed an integrated platform for dereplication and targeted analysis [8]. The metabolic profiles of more than 150 samples from *Laurencia* populations were analyzed using this approach in order to detect the presence of **1** and/or related secondary metabolites. Neorogioltriol (**1**) was successfully detected in a population of *Laurencia* sp. collected from the area of Vatsa bay in Kefalonia island, which produced mainly neorogioldiol (**2**) [7] and the related diterpene *O*^11^,15-cyclo-14-bromo-14,15-dihydrorogiol-3,11-diol (**3**) [7] (Figure 1).

**Figure 2:**
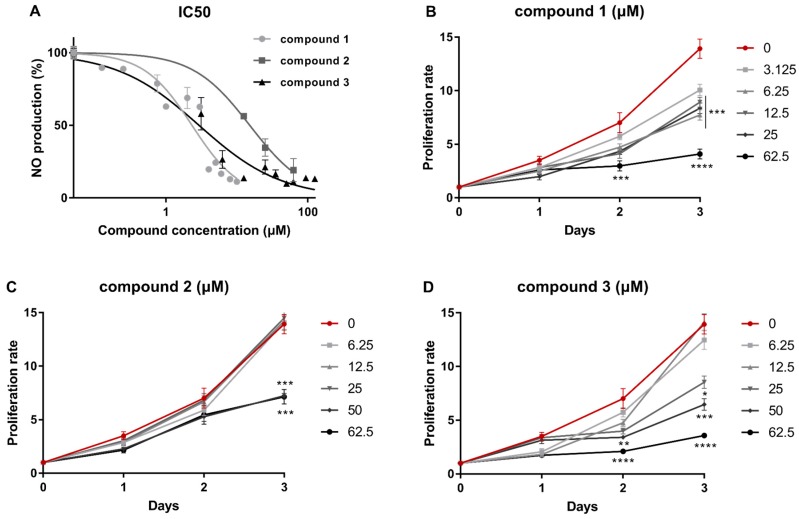
Determination of IC_50_ values and cytostatic potential of metabolites **1**–**3**. (**A**) Determination of the compound concentration resulting in 50% inhibition of NO production by RAW 264.7 cells. Evaluation of the effect of (**B**) neorogioltriol (**1**), (**C**) neorogioldiol (**2**), and (**D**) *O*^11^,15-cyclo-14-bromo-14,15-dihydrorogiol-3,11-diol (**3**) on the proliferation of RAW 264.7 cells. The cell number was determined using a Neubauer chamber and trypan blue staining, which was normalized to initial cells plated, and compared to cells treated with carbowax 400 0.1% *v/v*. Statistical analysis was carried out using the Kruskal-Walis non-parametric test in the Graphpad Prism 7.0 and graphs represent mean ± SEM (* indicates *P* < 0.05, ** indicates *P* < 0.01, *** indicates *P* < 0.001, **** indicates *P* < 0.0001).

Initially, **1**, **2,** and **3** were evaluated for their anti-inflammatory capacity by determining IC_50_ values in an in vitro macrophage-based nitric oxide production assay, as follows. RAW 264.7 were pre-treated with increasing concentrations of the respective compound for 1 h and then activated using 100 ng/mL LPS for 48 h. Nitric oxide (NO) was measured in the supernatant of the cell culture using Griess reaction and results were expressed as a percentage of NO production to the control including cells that were treated with the compound solvent carbowax 400 (Figure 2A). Neorogioltriol (**1**) exhibited the strongest anti-inflammatory capacity (IC_50_ = 2.32 ± 0.18 μM), which was followed by *O*^11^,15-cyclo-14-bromo-14,15-dihydrorogiol-3,11-diol (**3**) (IC_50_ = 2.92 ± 0.65 μM) and neorogioldiol (**2**) (IC_50_ of 15.16 ± 2.20 μM).

To determine whether reduction of NO production was due to the cytotoxity of the compounds, we measured the impact of increasing concentrations of the metabolites on RAW264.7 proliferation. The number of viable cells was determined using trypan blue exclusion on a Neubauer chamber during a three-day incubation period. None of the three compounds exhibited cytotoxicity below 62.5 μM during the first 24 h of incubation (Figure 2B–D). Neorogioltriol (**1**) showed significant cytostatic properties in concentrations above 6.25 μM three days post treatment (Figure 2B), in contrast to a previous report [6]. Metabolites **2** and **3** exhibited cytostatic effects at concentrations above 50 and 25 μM, respectively (Figure 2C,D), which suggests that lower concentrations would be optimal for therapeutic use.

### 2.2. Macrophages Exposed to Metabolites **1**–**3** Acquire an M2-Like Anti-Inflammatory Phenotype

Regulation of macrophage metabolism is tightly associated with macrophage physiology orchestrating their capacity to become activated, proliferate, and acquire an M1 phenotype [9,10,11]. Our results showed that the three investigated compounds exhibit anti-inflammatory activity and cytostatic effects, which suggests that they may impact macrophage function by either suppressing M1 polarization or promoting an anti-inflammatory M2 phenotype. In order to further study the effect of compounds **1**–**3** on macrophage M1/M2 phenotype and to determine their mechanism of action, naive RAW 264.7 macrophages were treated for 24 h with the compounds using a concentration approximately three times higher than their IC_50_ value, specifically, (**1**) 8 μΜ, (**2**) 62.5 μΜ, and (**3**) 10 μM. As a positive control, the diterpene sclareol, known for its anti-inflammatory capacity [12], was used in a concentration of 32.4 μΜ (equal to 10 μg/mL used in [12,13]).

**Figure 3:**
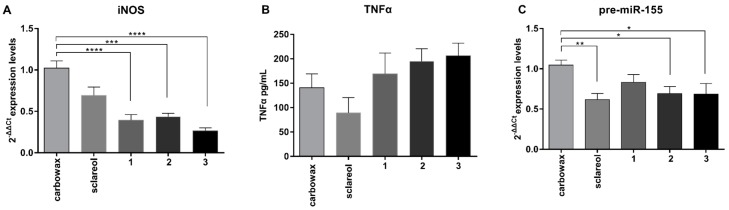
Effect of metabolites **1**–**3** on the expression of pro-inflammatory markers in RAW 264.7 cells following 24 h of incubation with the respective compounds. (**A**) iNOS mRNA levels measured using real time PCR, (**B**) TNFα production was measured using ELISA in the supernatant of cell culture, and (**C**) pre-miR-155 mRNA levels measured using real time PCR. All treatments have been compared to carbowax 400 0.1% *v/v* treated cells. Statistical analysis was carried out using Kruskal-Walis non-parametric test in Graphpad Prism 7.0 and graphs represent mean ± SEM (* indicates *P* < 0.05, ** indicates *P* < 0.01, *** indicates *P* < 0.001, **** indicates *P* < 0.0001).

The production of NO is regulated in macrophages by the inducible Nitric Oxide Synthase (iNOS). We measured the expression levels of iNOS in response to compound treatment and we found that, in all three cases, iNOS mRNA levels were down-regulated up to 40%, even in the absence of inflammatory stimulation (Figure 3A). Notably, no significant difference in TNFα production was observed (sclareol *P* = 1, (**1**) *P* = 1, (**2**) *P* = 0.4670, (**3**) *P* = 1) (Figure 3B). In addition, expression of the pro-inflammatory miRNA miR-155 was reduced following treatment with compounds **2** and **3** (Figure 3C). The effects of the metabolites at basal levels of pro-inflammatory mediators may have clinical significance in the context of Low Grade Systemic Inflammation (LGSI), such as the one observed during metabolic inflammation, where the metabolites may dampen LGSI.

**Figure 4:**
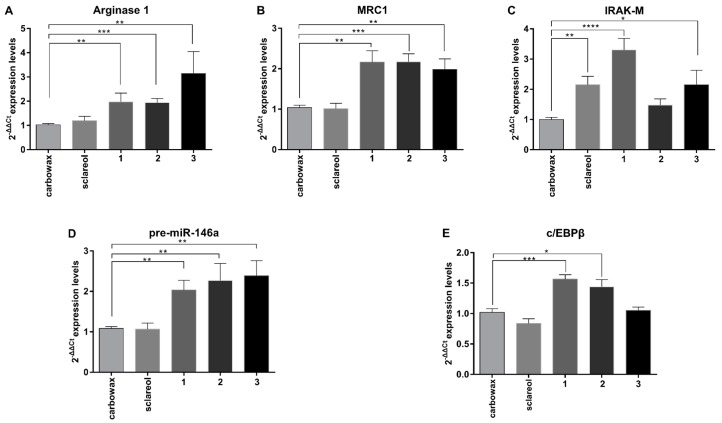
Effect of metabolites **1–3** on the levels of mRNA expression of anti-inflammatory markers in RAW 264.7 cells following 24 h of incubation with the respective compounds. (**A**) Arginase 1 mRNA levels, (**B**) MRC1 mRNA levels, (**C**) IRAK-M mRNA levels, (**D**) pre-miR-146a mRNA levels, and (**E**) c/EBPβ mRNA levels were measured using real time PCR. All treatments have been compared to carbowax 400 0.1% *v/v* treated cells. Statistical analysis was carried out using Kruskal-Walis non-parametric test in Graphpad Prism 7.0 and graphs represent mean ± SEM (* indicates *P* < 0.05, ** indicates *P* < 0.01, *** indicates *P* < 0.001, **** indicates *P* < 0.0001).

M2 type of macrophage activation is characterized by a distinct gene expression profile. Anti-inflammatory markers, including Arginase 1, MRC1, Ym1 and Fizz1, are induced by most M2-polarizing stimuli, such as prolonged LPS exposure. This results in endotoxin tolerance, IL-4, IL-13, and IL-10, which generates distinct M2-like phenotypes [4,14]. We, therefore, measured the M2 markers Arginase 1 and MRC1 in naive macrophages exposed to the three compounds and found that they were significantly up-regulated, which indicates that they all promoted an anti-inflammatory phenotype (Figure 4A,B). In addition, we measured the levels of the negative regulator of TLR signaling and M1 activation IRAK-M [15], and found that this was induced by both **1** and **3** (Figure 4C), which are the two metabolites with the most potent anti-inflammatory activity.

M2 macrophage polarization is regulated by members of the c/EBP family of transcription factors. Among them, c/EBPβ mediates TLR4 induced Arginase 1 expression and has been reported to promote the expression of several M2-regulating genes [16], such as MRC1 [17]. We measured c/EBPβ gene expression and found that it was significantly up-regulated in **1**-treated and **2**-treated RAW264.7 macrophages, whereas it was not affected in **3**-treated cells (Figure 4E). We have previously shown that c/EBPβ is a central regulator of anti-inflammatory macrophages [18] that controls the expression of IRAK-M and several miRNAs involved in macrophage activation [2,19,20]. Elevated levels of c/EBPβ in naive macrophages may indicate a state of reduced M1 activation capacity and a poised state to induce higher levels of anti-inflammatory genes following an appropriate stimulus [21].

We have previously shown that c/EBPβ controls miR-146a and miR-155, which are also induced upon activation to regulate TLR4 signaling in macrophages [19]. miR-146a is an anti-inflammatory miRNA implicated in the induction and maintenance of endotoxin tolerance [19] and the establishment of M2 phenotype in RAW 264.7 by promoting transcription of M2 genes, such as Arginase 1 and IL-10 [22]. In contrast, miR-155 is primarily involved in the initiation of inflammation and the induction of pro-inflammatory genes, which contributes to the M1 polarized phenotype and suppresses the induction of anti-inflammatory genes such as SOCS1 [23,24]. We examined the expression of the precursors of these two miRNAs in response to the compounds studied and found that miR-146a was up-regulated up to 2.5-fold compared to the control in response to all three compounds (Figure 4D), whereas miR-155 was significantly reduced when compared to the control (Figure 3C). Collectively, these findings suggest that diterpenes **1**–**3** utilize a similar mechanism of action promoting an anti-inflammatory M2-like phenotype in naive macrophages RAW 264.7 by inducing expression of anti-inflammatory mediators and reducing the expression of pro-inflammatory ones.

### 2.3. Metabolites **1–3** Suppress M1 Activation of Macrophages but do not Affect Induction of Endotoxin Tolerance

**Figure 5:**
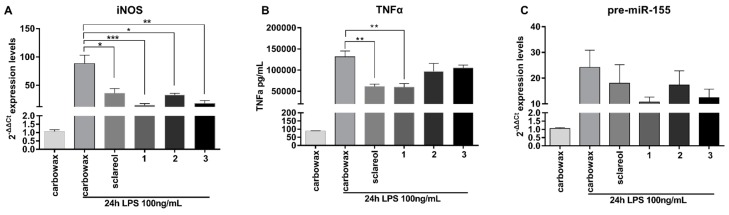
Evaluation of the pro-inflammatory potential of metabolites **1**–**3** upon LPS activation in RAW 264.7 cells. Cells were pre-incubated for 1 h with the respective compound, then co-incubated with LPS 100 ng/mL for 24 h. The expression profile of pro-inflammatory markers was evaluated. (**A**) iNOS and (**C**) pre-miR-155 mRNA levels were evaluated using real time PCR. (**B**) TNFα production was measured using ELISA in the supernatant of the cell culture. All treatments have been compared to carbowax 400 0.1% *v/v* treated cells. Statistical analysis was carried out using the Kruskal-Walis non-parametric test in Graphpad Prism 7.0 and graphs represent mean ± SEM (* indicates *P* < 0.05, ** indicates *P* < 0.01, *** indicates *P* < 0.001, **** indicates *P* < 0.0001).

To determine the effect of metabolites **1**–**3** in the production of inflammatory mediators from activated macrophages and the induction of endotoxin tolerance, we pre-treated RAW 246.7 macrophages for 1 h with the compounds and then stimulated cells with the TLR4 ligand LPS at a concentration of 100 ng/mL for 24 h. This type of stimulation triggers rapid induction of pro-inflammatory mediators, such as iNOS and TNFα, and subsequently promotes endotoxin tolerance by up regulating the M2 markers Arginase 1 and MRC1. To assess whether these compounds affect the intensity of inflammatory response to LPS, we measured the M1 gene markers iNOS and TNFα. All three compounds decreased iNOS expression up to 60% compared to the LPS-activated control sample (Figure 5A) in a similar trend, as seen in naive cells. In addition, secretion of TNFα in the cell culture supernatant was significantly lower in **1**-treated cells. However, the effect of **2**-treated and **3**-treated cells on TNFα secretion was not statistically significant (Figure 5B). Expression of the precursor of the pro-inflammatory miRNA miR-155 was not significantly lower in compound-treated cells (Figure 5C).

**Figure 6:**
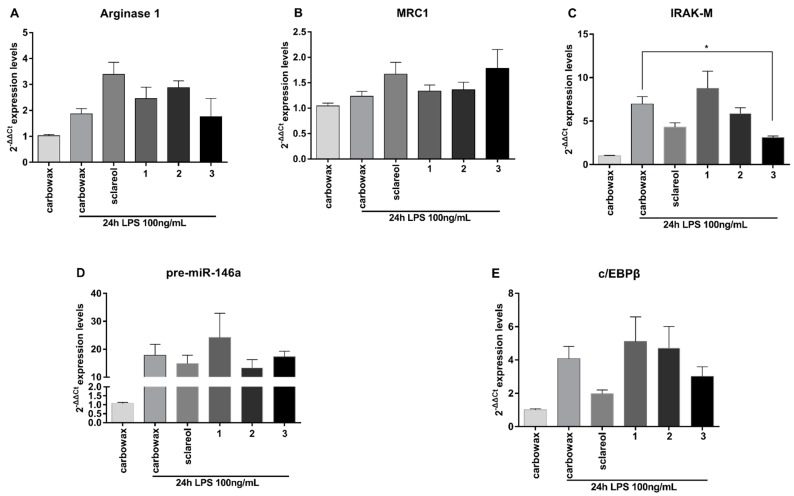
Evaluation of the anti-inflammatory potential of metabolites **1**–**3** upon LPS activation in RAW 264.7 cells. Cells were pre-incubated for 1 h with the respective compound and were then co-incubated with LPS 100 ng/mL for 24 h. Expression profile of anti-inflammatory markers was evaluated. (**A**) Arginase 1 mRNA levels, (**B**) MRC1 mRNA levels, (**C**) IRAK-M mRNA levels, (**D**) pre-miR-146a mRNA levels, and (**E**) c/EBPβ mRNA were measured using real time PCR. All treatments have been compared to carbowax 400 0.1% *v/v* treated cells. Statistical analysis was carried out using Kruskal-Walis non-parametric test in Graphpad Prism 7.0 and graphs represent mean ± SEM (* indicates *P* < 0.05, ** indicates *P* < 0.01, *** indicates *P* < 0.001, **** indicates *P* < 0.0001).

As expected, both M2 markers Arginase 1 and MRC1, were elevated when compared to the unstimulated control, but were not significantly different from the stimulated control (Figure 6A,B). Furthermore, IRAK-M (Figure 6C), pre-miR-146a (Figure 6D), and c/EBPβ (Figure 6E) were significantly up-regulated compared to naive control cells but compound-treated cells did not express significantly different levels of all genes tested compared to LPS stimulated controls, with the exception of **3,** which suppressed LPS-induced IRAK-M induction. We concluded that the function of metabolites **1**–**3** did not interfere with the induction of endotoxin tolerance in TLR4 mediated stimulated RAW 264.7 macrophages. Thus, these compounds suppressed LPS-induced activation of macrophages but did not affect the induction of endotoxin tolerance.

### 2.4. Evaluating the Effect of Metabolites **2** and **3** in DSS-Induced Colitis in Mice

**Figure 7:**
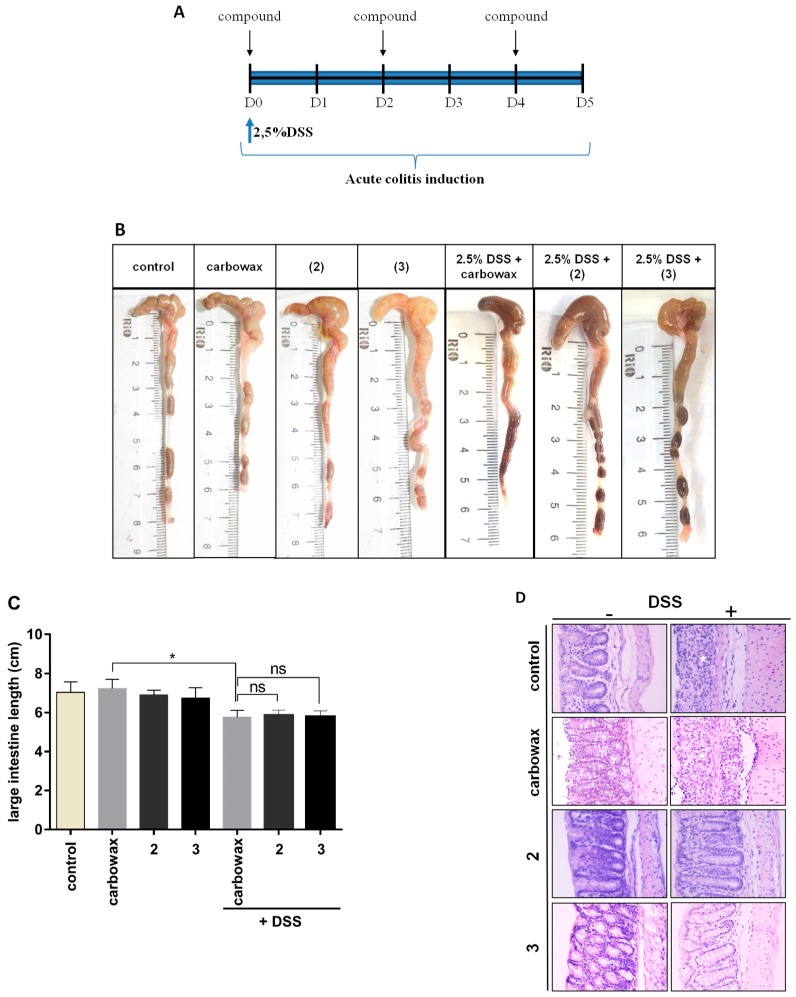

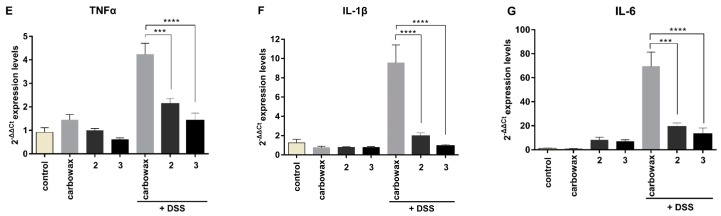
Identification of the potential in vivo anti-inflammatory properties of metabolites **2** and **3** using DSS-induced model of inflammatory bowel disease. (**A**) Schematic representation of the DSS protocol combined with intraperitoneal treatment of potential anti-inflammatory analogs. Female C57BL/6J mice received 2.5% in their drinking water for five days and were injected intraperitoneally with metabolites **2** and **3** every 48 h starting from day 0. “Control” mice received only drinking water. (**B**) Photos illustrating the length and the macroscopic view of a representative intestine of each group. (**C**) Graph with values of colon length of each group. (**D**) Hematoxylin and eosin staining of colon tissue sections from untreated (left panel) and DSS-treated mice (right panel) showing that treatment with **2** and **3** (lower right panels) ameliorates the disease phenotype (right upper vs. right lower images). Original magnification is x100. (**E**–**G**) Quantification of IL-6, IL-1b, and TNFα mRNA expression of groups indicated in the x-axis. Results were normalized to the housekeeping β-actin gene and expressed as RQ values relative to untreated controls, which was given the arbitrary value of ‘1’. Statistical analysis was carried out using Kruskal-Walis non-parametric test in Graphpad Prism 7.0 and graphs represent mean ± SEM (* indicates *P* < 0.05, ** indicates *P* < 0.01, *** indicates *P* < 0.001, **** indicates *P* < 0.0001).

Having evaluated the in vitro anti-inflammatory capacity of **1** and its congeners **2** and **3** in the cell culture, we proceeded to investigate their potential to suppress inflammation *in vivo*. For these studies, we utilized C57BL/6 mice, which carry M1-prone macrophages. The cell culture and in vivo results are comparable because RAW264.7 cells were derived from Balb/c background following oncogenic transformation and are also M1-prone. We used the model of Dextran Sodium Sulfate (DSS)-induced colitis in mice. DSS-induced colitis is a well-established and reproducible model widely used as a model of intestinal inflammation. It is characterized by bloody diarrhea, ulcerations, and infiltrations with granulocytes, which resemble features of flare-ups in human ulcerative colitis [25,26,27]. Since in vivo mice treatments require high amounts of purified compounds, low availability of the compounds allowed us to only perform these experiments with **2** and **3**. We used a five-day protocol where C57BL/6J mice received 2.5% DSS in their drinking water and were injected intraperitoneally with **2** and **3** every second day for this time period (Figure 7A). On day 6, mice were sacrificed, and intestines were isolated. Colon length has been proven to be a useful tool to ensure that DSS-treated mice respond to the pro-colitic agent [28] and is used as a measure of the severity of colonic inflammation. Length and the macroscopic view of representative colons from each group are shown in Figure 7B. All DSS-treated mice showed reduction of colon length, which confirms colonic inflammation macroscopically. Mice exposed to DSS had a significantly shorter colon, but treatment with the compounds did not alter its length (Figure 7C). Nevertheless, histological evaluation was performed using hematoxylin and eosin (H&E) staining in colon tissue sections, which shows that treatment with neorogioldiol (**2**) and *O*^11^,15-cyclo-14-bromo-14,15-dihydrorogiol-3,11-diol (**3**) reduced tissue damage by improving colonic histology after DSS treatment (Figure 7D). To investigate this finding, we quantified mRNA levels of pro-inflammatory cytokines in colon tissue. TNFα, which is the hallmark cytokine of colitis, was found to be significantly reduced in both **2**-treated and **3**-treated mice (Figure 7E). Next, we measured mRNA levels of the pro-inflammatory cytokines IL-1β and IL-6, which are major determinants of colitis [27]. IL-1β was significantly reduced in both **2**-treated and **3**-treated mice (Figure 7F). Similarly, **2**-treated and **3**-treated mice exhibited more than 40-fold decrease in IL-6 mRNA levels whereas basal levels of IL-6 in **2**- and **3**- but not DSS-treated mice were not significantly elevated (**2**, *P* = 1, **3**, *P* = 1) (Figure 7G).

To determine the systemic cytotoxicity of the compounds, we histologically examined the liver of mice treated with **2**, as well as the serum levels of liver enzymes and tissue damage markers. No significant cytotoxicity was observed in treated mice, as indicated by histology and serum levels of ALT, AST, LDH, and CPK (Figure S1). Taken together, these findings suggest that **2** and **3** have strong in vivo anti-inflammatory properties, which suppresses colon inflammation and tissue damage in the model of DSS-induced colitis.

## 3. Materials and Methods

### 3.1. General Experimental Procedures

Optical rotations were measured on a Perkin Elmer model 341 polarimeter (PerkinElmer Instruments, Norwalk, CT, USA) with a 1 dm cell. UV spectra were obtained on a Perkin Elmer Lambda 40 spectrophotometer (PerkinElmer Ltd., Buckinghamshire, UK). IR spectra were obtained on a Bruker Tensor 27 spectrometer (Bruker Optik GmbH, Ettlingen, Germany). NMR spectra were recorded on Bruker AC 200 and Bruker DRX 400 spectrometers (Bruker BioSpin GmbH, Rheinstetten, Germany). The 2D NMR experiments (HSQC, HMBC, COSY) were performed using standard Bruker pulse sequences. High-resolution ESI mass spectra were measured on a Thermo Scientific LTQ Orbitrap Velos mass spectrometer (Thermo Fisher Scientific, Bremen, Germany). Low-resolution EI mass spectra were measured on a Thermo Electron Corporation DSQ mass spectrometer (Thermo Electron Corporation, Austin, TX, USA) using a Direct-Exposure Probe (Thermo Electron Corporation, Austin, TX, USA). Normal-phase column chromatography separations were performed with Kieselgel Si 60 (Merck, Darmstadt, Germany). HPLC separations were conducted on a Pharmacia LKB 2252 liquid chromatography pump (Pharmacia LKB Biotechnology, Uppsala, Sweden) equipped with a RI-102 Shodex refractive index detector (ECOM spol. s r.o., Prague, Czech Republic) using an Econosphere Silica 10 μm (250 × 10 mm i.d.; Grace, Columbia, MD, USA) column. TLC was performed with Kieselgel 60 F_254_ aluminum plates (Merck, Darmstadt, Germany) and spots were detected after spraying with 15% H_2_SO_4_ in MeOH reagent and heating at 100 °C for 1 min.

### 3.2. Collection of Algal Material

Specimens of *Laurencia* sp. were collected by hand at Vatsa bay in Kefalonia island, Greece, at a depth of 0.5 to 2 m in May 2014. A voucher specimen of the alga was deposited at the Herbarium of the Section of Pharmacognosy and Chemistry of Natural Products, Department of Pharmacy, National and Kapodistrian University of Athens (ATPH/MP0444).

### 3.3. Isolation of Metabolites **1**–**3**

Specimens of the fresh alga were exhaustively extracted with mixtures of CH_2_Cl_2_/MeOH at room temperature. Evaporation of the solvents in vacuo afforded a dark green oily residue (27.3 g) that was subjected to vacuum column chromatography on silica gel, using cyclohexane with increasing amounts of EtOAc, which is followed by EtOAc with increasing amounts of MeOH as the mobile phase, to yield 17 fractions (1–17). Fractions 5 (30% EtOAc in cyclohexane) and 6 (35% EtOAc in cyclohexane) were combined (6.1 g) and fractionated by gravity column chromatography on silica gel, using cyclohexane with increasing amounts of EtOAc as the mobile phase, to afford a fraction (10% EtOAc in cyclohexane) that was further purified by normal-phase HPLC, using cyclohexane/EtOAc (95:5) as eluent, to yield **3** (578.4 mg). Fractions 9 (50% EtOAc in cyclohexane), 10 (55% EtOAc in cyclohexane), and 11 (60% EtOAc in cyclohexane) were combined (3.3 g) and fractionated by gravity column chromatography on silica gel, using cyclohexane with increasing amounts of EtOAc as the mobile phase, to afford a fraction (15% EtOAc in cyclohexane) that was further purified by normal-phase HPLC, using cyclohexane/Me_2_CO (85:15) as eluent, to yield **2** (682.0 mg). Fractions 15 (100% EtOAc) and 16 (10% MeOH in EtOAc) were combined (2.6 g) and fractionated by gravity column chromatography on silica gel, using CH_2_Cl_2_ with increasing amounts of Me_2_CO, which was followed by Me_2_CO with increasing amounts of MeOH as the mobile phase, to yield 10 fractions (15a–15j). Fraction 15d (80% Me_2_CO in CH_2_Cl_2_, 138.3 mg) was purified by normal-phase HPLC, using cyclohexane/EtOAc (40:60) as eluent, to yield **1** (12.1 mg).

### 3.4. Cell Culture

The mouse macrophage cell line RAW 264.7 was obtained from LGC standards and used previously in our laboratory [19,20]. RAW 264.7 cells were cultured in DMEM medium (cat. # 21885-025, Gibco) supplemented with 10% (*v/v*) heat inactivated fetal bovine serum (cat. # 10270-106, Gibco) and 1% (*v/v*) penicillin-streptomycin (cat. # 15070-063, Gibco). Cells were grown in 37 °C and 5% CO_2_. Each compound used was diluted in Carbowax^TM^ 400 + 10% (*v/v*) absolute ethanol (cat. # 1.00983, Sigma) serving as a control solvent. The final concentration in culture was 0.1% (*v/v*) carbowax and 0.01% (*v/v*) ethanol. Macrophage activation was performed using 100 ng/mL lipopolysaccharide (LPS) (L2630, Sigma) and, in the case of compound treated cells, macrophages were pre-treated for 1 h with the respective compound before LPS stimulation. Compound concentrations used for treatment were approximately three times higher than the IC_50_ value of each compound, specifically, (**1**) 8 μΜ, (**2**) 62.5 μΜ, and (**3**) 10 μM.

### 3.5. Cell Proliferation Assay

Macrophages were seeded in 96-well plates and treated with various concentrations of the respective compounds. After 24, 48, and 72 h of treatment, cells were scrapped, dyed with trypan blue, and the viable cell number was measured in a Neubauer chamber.

### 3.6. Measurement of Nitrite Production

Furthermore, 25 × 10^4^ RAW 264.7 macrophages per sample were plated in 24 well plates with 0.5 mL DMEM and pretreated for 1 h with the indicated concentrations of compounds **1**–**3**. Then, the cells were incubated with 100 ng/mL LPS for 48 h. The amount of nitrite, an oxidative product of NO, was measured in the supernatant of each culture using Griess reaction. Additionally, 50 μL of the supernatant was mixed with 50 μL of sulfanilamide solution (1% sulfanilamide in 5% H_3_PO_4_) and incubated for 5 min at room temperature. Then, 50 μL of NED solution (0.1% *N*-1-napthylethylenediamine dihydrochloride in H_2_O) was added and the absorbance was measured in an automated microplate reader (Infinate 200 PRO, Tecan, Männedorf, Switzerland) at 540 nm. Nitrite concentration was calculated using a sodium nitrite standard curve and all incubations were performed in the dark.

### 3.7. cDNA Synthesis and Quantitative PCR for Cell Culture Samples

For each sample, 25 × 10^4^ cells were seeded in a 24-well plate with 0.5 mL DMEM. Cells were treated with the appropriate compound and activated using LPS for 24 h. Wells were washed with ice-cold PBS and cells were harvested using the Trizol reagent (Invitrogen, 15596026). RNA isolation was carried out according to the manufacturer’s instructions. Reverse transcription to 1 μg of total RNA per sample was performed using PrimeScript™ 1^st^ strand cDNA synthesis Kit (Takara, 6110A) and random 6-mer primers, according to manufacturer’s instructions. Each sample was diluted three times and used as a template in duplicate for two-step quantitative PCR in a 7500 Fast Real-Time PCR Instrument (Applied Biosystems^®^, 4351106) with 96-well Block Module as follows: start step 95 °C for 3 min and 40 cycles of 95 °C for 10 s and 60 °C for 20 s. Amplification was performed using KAPA SyBr^®^ Fast Universal qPCR kit (Kapa Biosystems, KK4618). The primers used are listed in Supplementary Table S1. Data analysis was accomplished using mRNA levels expressed as relative quantification (RQ) values, which were calculated as RQ = 2^(−ΔΔCt)^, where ΔCt is (Ct (gene of interest) − Ct (housekeeping gene)). *rsp9* mRNA was used as the internal control gene.

### 3.8. Elisa

Cytokine concentration for mouse TNFα in the medium of activated macrophages was determined using the Mouse TNFα Elisa Max™ Delux Set (BioLegent, 430904) following the manufacturer’s instructions.

### 3.9. Mice

C57BL/6 mice were maintained in identical conditions prior to treatments. For the acute colitis model, mice (20–22 g weight) were administered 2.5% (*w/v*) DSS in water for five consecutive days. For the assessment of the effects of compounds **2** and **3**, each compound was dissolved in carbowax 400 and administered intraperitoneally at a dose of 0.25 mg/mouse every 48 h starting from day 0. Typically, three to five mice were used per group and each experiment was performed at least twice. All procedures were conducted in compliance with protocols approved by the Animal Care Committee of the University of Crete, School of Medicine (Facility number EL91-BIOexp-06) and the Institute of Molecular Biology and Biotechnology-FORTH (Facility Number EL91-BIOexp-02), Heraklion, Crete, Greece, in conjunction with the Veterinary Department of the Region of Crete, Heraklion, Crete, Greece (license numbers 27298 and 269884).

### 3.10. Tissue Processing and Histological Evaluations

For histopathology, the heart and the large intestine of the mouse was removed, opened longitudinally, fixed in buffered formalin, and embedded in paraffin for histological analyses. Sections of 5 μM were prepared, placed on glass lesions, and stained with hematoxylin and eosin (H&E) to assess inflammatory infiltration and tissue damage.

### 3.11. Determination of Biochemical Markers

To detect serum levels of alanine aminotransferase (ALT) and aspartate aminotransferase (AST) activity, creatine phosphokinase (CPK) and lactate dehydrogenase (LDH) serum was collected from peripheral blood via cardiac puncture under isoflurane anesthesia. Serum samples were stored at −20 °C until it was ready to use. The levels of ALT, AST, CPK, and LDH were assayed by standard enzymatic procedures using an automated biochemical analyzer ADVIA (Siemens) in the Laboratory of Clinical Biochemistry, University Hospital of Heraklion (Crete, Greece).

### 3.12. Real-Time PCR from Mouse Tissue Samples

RNA was isolated from large intestine or liver tissue using the Nucleospin RNA kit (MACKEREY-NAGEL). Total RNA was quantified with a Nanodrop Spectrophotometer. A High-Capacity complementary DNA Reverse Transcription kit was used to synthesize cDNA from 500 ng RNA, according to the manufacturer’s protocol using the High-Capacity cDNA Archive kit (Applied Biosystems). Applied Biosystems TaqMan Universal PCR Mastermix and TaqMan gene expression probes for mouse IL-6 (ID Mm00446190_m1, FAM), TNF (ID Mm00443259_g1, FAM), IL-1b (ID Mm00434228_m1, FAM), and β-actin (ID Mm00607939_s1, VIC labeled) as an endogenous control from Applied Biosystems and used on an Applied Biosystems ViiA Real-Time PCR Instrument. All assays were run in duplicate on an Applied Biosystems ViiA Real-Time PCR system, according to the manufacturer’s instructions, and the mean value was used for the analysis. mRNA levels were expressed as relative quantification (RQ) values, which were calculated as RQ = 2^(−ΔΔCt)^, where ΔCt is (Ct (gene of interest) − Ct (housekeeping gene)).

### 3.13. Statistical Analysis

All data are presented as mean ± SEM, from at least three independent experiments, and as a percentage in the case of IC_50_ evaluation. Statistical analysis was performed using Graphpad Prism 7.0 (GraphPad Software, San Diego, CA). D’Agostino & Pearson, Shapiro Wilk and KS tests were used to evaluate normality. In case of normality, one-way ANOVA was performed, whereas, in all other cases, the non-parametric Kruskal-Wallis test was used. Differences with a *P* value < 0.05 are considered significant (* indicates *P* < 0.05, ** indicates *P* < 0.01, *** indicates *P* < 0.001, **** indicates *P* < 0.0001).

## 4. Conclusions

Based on these findings, we can conclude that neorogioltriol (**1**) and its related compounds neorgioldiol (**2**) and *O*^11^,15-cyclo-14-bromo-14,15-dihydrorogiol-3,11-diol (**3**) promote an anti-inflammatory, M2-like phenotype in macrophages by inducing the expression of multiple molecules affecting macrophage polarization. As a result, the response to pro-inflammatory stimuli, such as LPS, is reduced. The anti-inflammatory action of compounds (**2**) and (**3**) was confirmed in vivo in the model of colitis, which suggests a potential of these molecules as leads for the development of anti-inflammatory molecules targeting macrophage polarization mechanisms.

## Supplementary Materials

The following are available online at http://www.mdpi.com/1660-3397/17/2/97/s1, Table S1: List of oligonucleotides used in real time PCR reactions, Figure S1: Identification of the potential in vivo cytotoxic properties of metabolite **2** in heart and liver tissue, Figure S2. ^1^H NMR spectrum (400 MHz, CDCl_3_) of neorogioltriol (**1**), Figure S3. ^1^H NMR spectrum (400 MHz, CDCl_3_) of neorogioldiol (**2**), Figure S4. ^1^H NMR spectrum (400 MHz, CDCl_3_) of *O*^11^,15-cyclo-14-bromo-14,15-dihydrorogiol-3,11-diol (**3**).
