# Supplementary material for: Neorogioltriol and Related Diterpenes from the Red Alga Laurencia Inhibit Inflammatory Bowel Disease in Mice by Suppressing M1 and Promoting M2-Like Macrophage Responses
Source: Mar Drugs. 2019 Feb 2;17(2):97. doi: 10.3390/md17020097 (PMC6410277; doi:10.3390/md17020097)
Supplement: Supplementary file 1 [file marinedrugs-17-00097-s001.pdf]

Supplementary materials

Neorogioltriol and Related Diterpenes from the Red Alga *Laurencia* Inhibit Inflammatory Bowel Disease in Mice by Suppressing M1 and Promoting M2-Like Macrophage Responses

Maria G. Daskalaki [1,2,3], Dimitra Vyrla [3], Maria Harizani [4], Christina Doxaki [2,3], Aristides G. Eliopoulos [3,5], Vassilios Roussis [4], Efstathia Ioannou [4,*], Christos Tsatsanis [2,3,*] and Sotirios C. Kampranis [1,6,*]

[1] Laboratory of Biochemistry, School of Medicine, University of Crete, Heraklion 70013, Greece; m.daskalaki@med.uoc.gr (M.G.D.)

[2] Laboratory of Clinical Chemistry, School of Medicine, University of Crete, Heraklion 70013, Greece; cdoxaki@med.uoc.gr

[3] Institute of Molecular Biology and Biotechnology, FORTH, Heraklion, Crete 71110, Greece; di_micro@hotmail.com (D.V.); eliopag@med.uoa.gr (A.G.E.)

[4] Section of Pharmacognosy and Chemistry of Natural Products, Department of Pharmacy, National and Kapodistrian University of Athens, Panepistimiopolis Zografou, Athens 15771, Greece; mariachariz@pharm.uoa.gr (M.H.); roussis@pharm.uoa.gr (V.R.)

[5] Present address: Laboratory of Biology, School of Medicine, National and Kapodistrian University of Athens, Athens 11527, Greece

[6] Present address: Section of Plant Biochemistry, Department of Plant and Environmental Sciences, University of Copenhagen, Thorvaldsensvej 40, 1871 Frederiksberg C, Denmark

* Correspondences: eioannou@pharm.uoa.gr (E.I.); tsatsani@uoc.gr (C.T.); soka@plen.ku.dk (S.C.K.); Tel.: +30-210-727-4913 (E.I.); +30-693-710-7708(C.T.); +30-697-221-3616 (S.C.K.)

Table S1. List of oligonucleotides used in real time PCR reactions

**Primer**	**Sequence**
rsp9-Fwd	GCTAGACGAGAAGGATCCCC
Rsp9-Rev	CAGGCCCAGCTTAAAGACCT
Pri-miR-155-Fwd	ACCCTGCTGGATGAACGTAG
Pri-miR-155-Rev	CATGTGGGCTTGAAGTTGAG
Pri-miR-146a-Fwd	CACGGACCTGAAGAACACTGG
Pri-miR-146a-rev	AGAAATGAAATTAGAACACACATCAATCC
Arginase 1-Fwd	CAGAAGAATGGAAGAGTCAG
Arginase 1-Rev	CAGATATGCAGGGAGTCACC
MRC1-Fwd	TTTCCATCGAGACTGCTGC
MRC1-Rev	ACCAAAGCCACTTCCCTTC
IRAK-M-Fwd	TTCCTGGCACGTTCGAATCA
IRAK-M-Rev	CGCTGCAGCAAAATCCGTTA
c/EBPβ-Fwd	GGGGTTGTTGATGTTTTTGGTT
c/EBPβ-Rev	TCACTTTAATGCTCGAAACGGA
iNOS-Fwd	TCCTGGAGGAAGTGGGCCGAAG
iNOS-Rev	CCTCCACGGGCCCGGTACTC

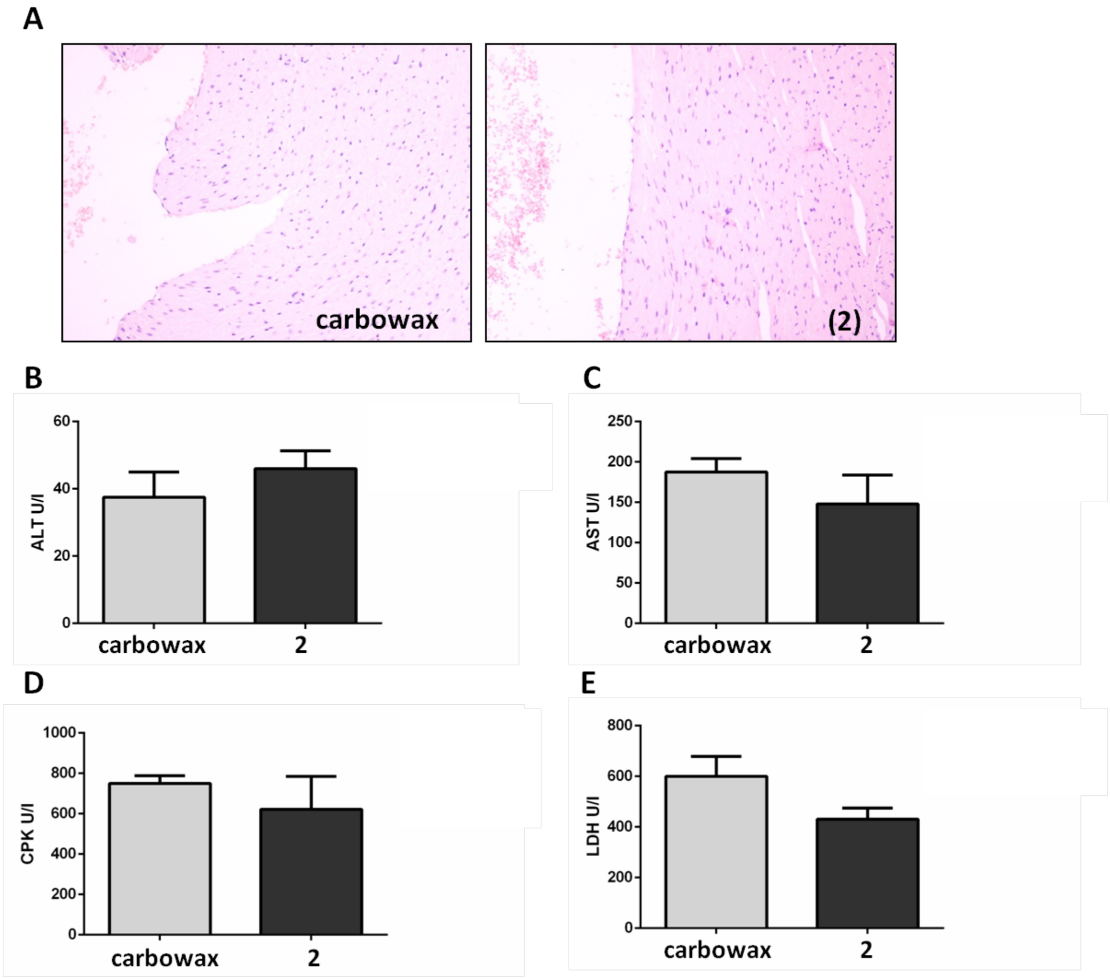

Figure S1. Identification of the potential *in vivo* cytotoxic properties of metabolite **2** in heart and liver tissue. (**A**) Hematoxylin and eosin (H&E) staining of heart tissue sections from untreated (left panel) and **2**-treated mice (right panel) showing normal appearing ventricle myocardium in both carbowax and **2**- treated mouse. Original magnification is x200. (**B-E**) Analysis of biochemical markers ALT, AST, CPK and LDH showing that treatment with **2** does not induce any unwanted liver or heart toxicity.

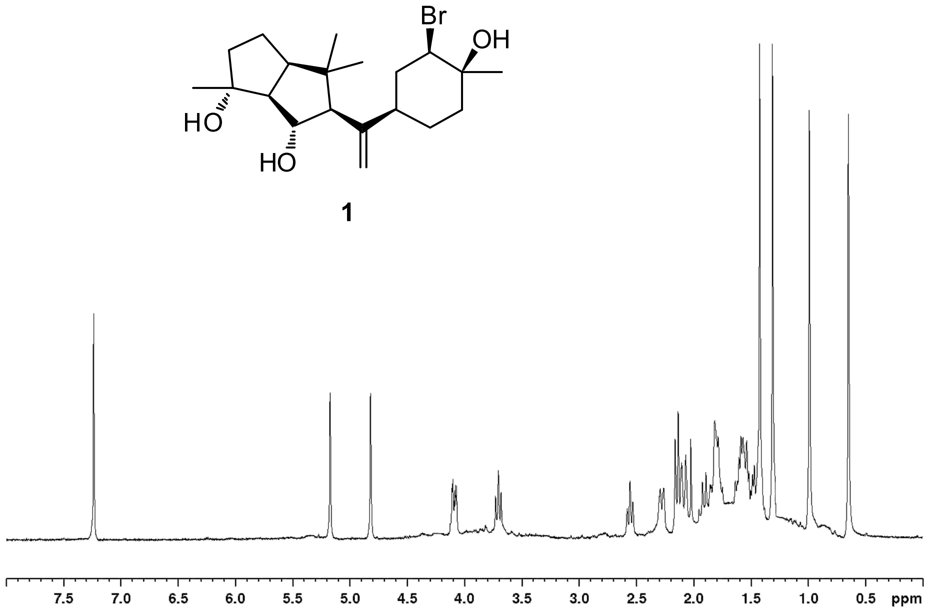

Figure S2. ^{1}H NMR spectrum (400 MHz, $CDCl_3$) of neorogioltriol (**1**).

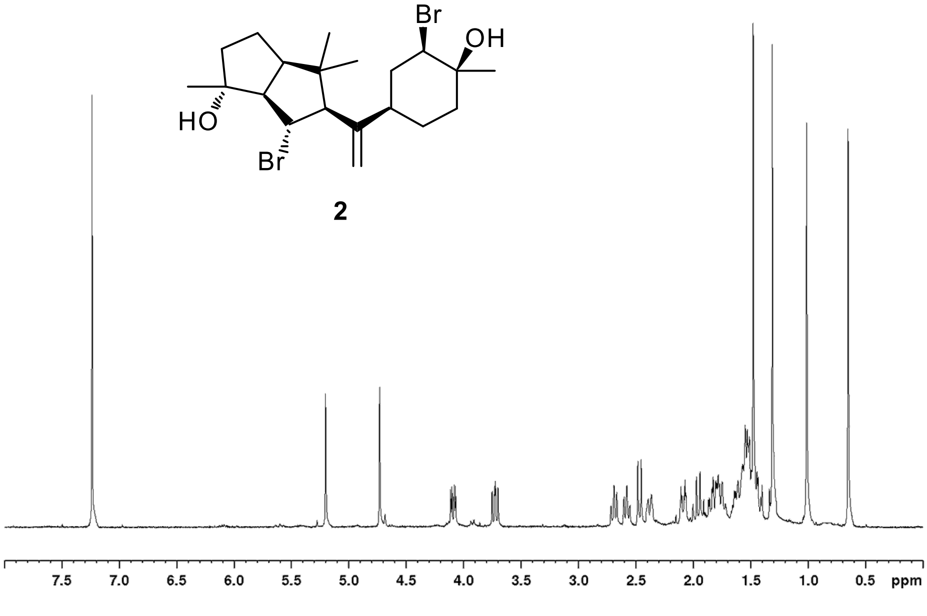

Figure S3. ^{1}H NMR spectrum (400 MHz, $CDCl_3$) of neorogioldiol (**2**).

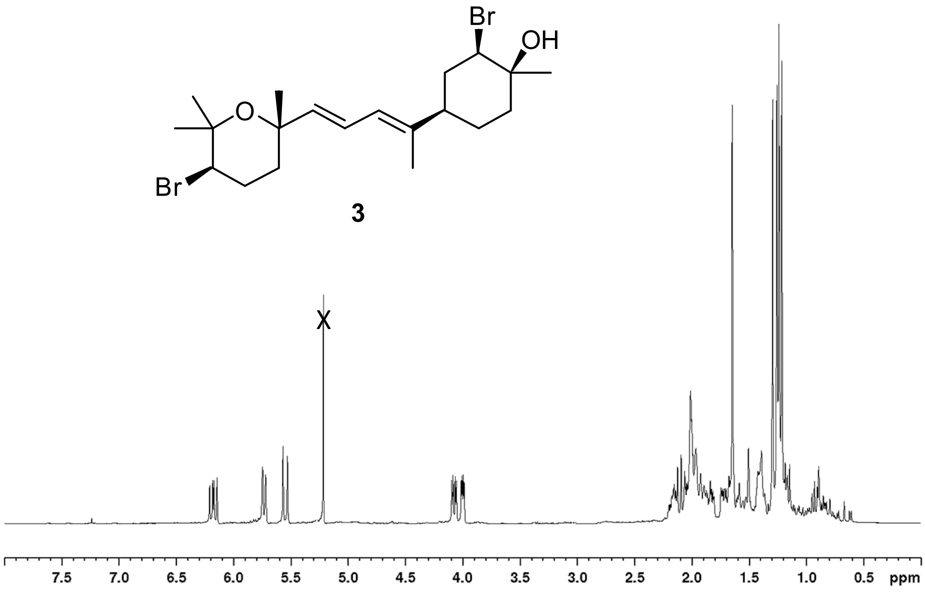

Figure S4. ^{1}H NMR spectrum (400 MHz, $CDCl_3$) of *O*^{11},15-cyclo-14-bromo-14,15-dihydrorogiol-3,11-diol (**3**).
